# An in-depth exploration of researcher experiences of time and effort involved in health and social care research funding in the UK: The need for changes

**DOI:** 10.1371/journal.pone.0291663

**Published:** 2023-09-21

**Authors:** Katie Meadmore, Hazel Church, Ksenia Crane, Amanda Blatch-Jones, Alejandra Recio Saucedo, Kathryn Fackrell

**Affiliations:** National Institute for Health and Care Research (NIHR) Coordinating Centre, School of Healthcare Enterprise and Innovation, University of Southampton, Southampton, United Kingdom; University of Nigeria, NIGERIA

## Abstract

The need to reform the way in which research is undertaken is clear, with reducing research bureaucracy and waste at the forefront of this issue for the UK government, funding organisations, higher education institutions and wider research community. The aim of this study was to describe researchers’ experiences of the time, effort and burden involved in funding processes–namely applying for research funding and fulfilling reporting requirements. This was an in-depth qualitative study using semi-structured interviews with researchers who had experience applying for funding and/or completing reporting requirements for a UK health and social care research funder between January 2018 and June 2021. Following thematic analysis, five key themes were identified describing researcher experiences of key issues around time, efforts and burden associated with funding processes. These themes encompassed (1) issues with the current funding model for health and social care research, (2) time and effort involved in funding processes, (3) the need for a streamlined end-to-end process, (4) implications for work-life balance, and (5) addressing the need for better support and communication. The findings from this study describe researcher experiences of tasks in the research pathway that currently take considerable time and effort. It was clear that whilst some of this time and effort is considered necessary, some is exacerbated by inefficient and ineffective processes, such as perceived under-funding of research or lack of clarity with regards to funder expectations. This in turn contributes to unnecessary researcher burden, research waste and negative research culture. Better investment in health and social care research and in the researchers themselves who design and deliver the research, alongside improvements in transparency, streamlining and research support could ensure a more positive research culture, and improve the quality of funded research.

## Introduction

In recent years researchers are finding themselves with ever-changing job roles, more activities being added to workloads and expectations that these are incorporated alongside their research work. For example, a 2021 survey from the University and College Union in the UK indicated that 81% of research only or teaching and research staff in Higher Education Institutes (HEIs) felt that their overall workload had increased over the last three years [1]. Staff identified increased administrative work, widening duties and increased online working as being top contributors to changes in their workload. Similarly, Rule and LeGouill [2] reported a perceived significant increase in the number of additional tasks and activities that different organisations required of teams for clinical research to be undertaken, such as multiple amendments, re-consent, and compliance processes.

In an ever-competitive environment for career progression and job security, researchers also often find themselves striving to build good research reputations by having to continually produce publications and other research outputs, demonstrate the impact of their research (including how their findings have been implemented, disseminated and benefit health and social care patients or services) and secure research funding. However, it is recognised that these activities contribute to high levels of burden [3–6]. For example, it has been estimated that writing a research funding application can take anywhere from 116 hours to 38 working days [3, 7, 8], and only about 20% of researchers who apply for funding are successful in securing research funds (e.g., [9]). For those who are awarded research funding, success then brings further post award administrative tasks (such as setting up contracts, hiring staff and getting ethical approvals), as well as reporting and monitoring requirements (such as progress reports, end of research project reports, or recruitment updates) and navigating implementation, dissemination and impacts of research. All of these additional tasks take a significant proportion of a researchers’ available working time, and whilst some are indeed necessary and relevant to research, especially when using public funds, others are felt to be too bureaucratic, wasting effort and taking time away from conducting the research itself. Unsurprisingly this has led to pressures on researchers’ job satisfaction, productivity, work-life balance and well-being [10–13].

The need to reform processes along the research pathway is clear, with reducing research bureaucracy and waste at the forefront of this issue [13–17]. In the UK, in the last few years, a number of governmental reports and reviews have raised concerns about research bureaucracy and burden and how this is stifling the UK research environment, both in terms of productivity and culture [14, 15, 18]. For example, a recent review highlighted key issues in existing research funding mechanisms and systems, including the need for greater alignment of processes and systems involved in applying for funding and management of research funds successfully awarded [15]. However, whilst the review provides a good, broad oversight of potential areas in which research bureaucracy could be changed within the sector, there is still limited evidence available on the specific needs of researchers, what they perceive as effortful and burdensome and the effects that this has on them and their research.

To address this gap, this study aimed to explore in-depth and build better understanding of researchers’ experiences of the processes, effort and burden involved in the funding system–namely preparing and applying for funding, and fulfilling reporting requirements for a UK health and/or social care research funding organisation between January 2018 and June 2021. Specifically, the research question posed was ‘what do researchers perceive as necessary and unnecessary processes, effort and burden in research funding’? This study was part of a two-phased project. Phase 1 was an online survey conducted with 183 researchers to begin to build knowledge on the types of effort and burden experienced by researchers and the processes associated with it (see [19]). The findings from Phase 1 were used to inform Phase 2 (this study) protocol and interview topic guides to further explore researchers’ experiences of the perceived effort and burden involved in their funding and research activities. This paper reports the second (interview) phase of this work.

## Methods

### Qualitative approach

This study was conducted as part of the National Institute for Health and Care Research (NIHR) research on research programme of work based at the Southampton Coordinating Centre, University of Southampton. The study uses a phenomenological qualitative approach to explore and understand researchers’ experiences of applying for and/or completing monitoring and reporting requirements for research funding in the UK. We took a relativism ontological position, understanding that researchers’ perceptions of their research experiences would be directly affected by consequences of research activities undertaken and the interactions they had with others (e.g., researchers, funders, editors, public). The study received ethical approval from the Faculty of Medicine, University of Southampton ethics committee (id 64868/64868.A1). Following online written informed consent from participants, data were collected using semi-structured interviews. The study is reported using the COREQ checklist for reporting qualitative data [20].

### Recruitment

Participants were researchers who had completed or contributed towards an application submission and/or fulfilled monitoring and reporting requirements for any health and/or social care research from a UK funding organisation during January 2018 to June 2021. These researchers had previously completed a survey on the same topic [19] and at the end of the survey, 68 researchers self-identified as being interested in taking part in follow-up interviews. From this list, purposeful sampling by career stage was used to ensure participants across career stages were invited (via email invitation) to take part in the interviews. Participants were sent study information (aims and details of study) and asked to complete the consent and demographics forms through an access link, if they were still interested in taking part in the interviews. Those who completed the form were then contacted to arrange a convenient time and date to participate in an interview. Invitations were sent to 10 participants at a time to manage responses and until a sample of at least 25 were recruited. Participants were recruited between March and May 2022.

### Reflexivity statement

The authors all work for the NIHR on the Research on Research programme of work and are based at the Southampton Coordinating Centre, University of Southampton. The authors are female and comprise research fellows and an assistant research manager. Four authors (KM, ABJ, ARS, KF) have over 10 years of qualitative research experience. All authors have experience with applying for and reporting on research funding, and have expert knowledge of funding mechanisms (although specific backgrounds and experiences are different). Although one interviewee was known to the research team from other projects, all authors were careful not to bias interview questions or overinterpret the data based on personal experiences and knowledge of funding processes. This was achieved through continuous monitoring and checks with the team and wider colleagues.

### Interview guide

A semi-structured interview topic guide was developed to aid discussion and allow for a comprehensive exploration of the researchers’ experiences with applying for funding and/or fulfilling monitoring and reporting requirements. The topic guide was developed using the findings from the Phase 1 survey exploring the same topic [19], and was split into three main sections: (i) the application process, (ii) the monitoring and reporting process, and (iii) the overall burden experienced (see S1 Table). The topic guide was reviewed by members of the NIHR and members of the team working on [15] to ensure that the questions were relevant to funders and the wider research sector and remained objective, generalisable and trustworthy. Following the first seven interviews, the topic guide was reviewed and refined in light of the initial themes coming from ongoing data analysis and to ensure that all aspects of experiences were captured. Changes were agreed by all interviewers.

### Data collection

Qualitative data were generated using semi-structured interviews conducted between March and May 2022. Interviews were conducted by one of three researchers (KM, KC and HC) and took place online using Microsoft Teams. Interviews lasted between 23 minutes and 1 hour 31 minutes (mean = 58 minutes). Interviews were recorded on MS Teams and the audio recording was transcribed by an external company to enable data analysis. Each interviewer checked transcripts against their audio recordings to ensure accuracy. Although a semi-structured approach allowed for interviewers to follow natural flows and avenues of conversation, the topic guide helped reduce potential interviewer bias by ensuring that certain questions were asked and in a similar way. All interviews were reviewed by KM. All interviewers had access to information that could identify the interviewee.

### Data analysis

All interview data was analysed using thematic analysis [21]. This allowed identification of the key efforts and burdens associated with applying for researching funding and reporting requirements as well as any associated issues or consequences. Data analysis started after the first interviews were transcribed and checked for accuracy (i.e. alongside data collection). This was to ensure that the topic guide was adequately eliciting responses on researchers’ experiences, and to monitor data saturation. The coding process was inductive as themes were data driven and no prior thematic framework was considered, and codes were not grouped under areas highlighted in the interview guide.

All transcripts were anonymised and imported into NVivo. Three researchers (KM, KC and HC) listened back to the recordings, read the interview transcripts and generated initial codes independently. All three researchers then met to discuss initial codes, identify themes, and agree on an initial thematic framework. To ensure consistency of coding, one author (KM) coded all of the data in NVivo using the framework. Following this, and to ensure that the themes were a true reflection of the data, all coded data in the themes were reviewed, revised and refined through discussions with the research team. This was an iterative process first conducted between the three interviewers (KM, KC and HC) to ensure the themes reflected the tone of the interviews. KF then independently reviewed the themes (to ensure the themes were data driven), before the team met to finalise the themes. Any disagreements in code placement or theme/subtheme names were discussed and agreed by consensus. Descriptions of the findings from each overarching theme and subtheme were then created and reviewed by all authors.

## Results

### Participant characteristics

Of the 68 researchers who indicated an interest in being interviewed, a total of 55 were contacted and invited to take part in the interview study. From this, 29 researchers agreed to take part; however, two were not interviewed (one withdrew due to other priorities, another did not respond with their availability). Thus, a total of 27 researchers (13 female (48%); 14 male (52%)) were interviewed (henceforth called participants), of which most identified as White and/or British (20, 74%), and the remaining seven identified as White-European, White other, Scottish, mixed, British Indian or Chinese (numbers not reported to maintain confidentiality). Most participants were senior researchers (17, 63%), followed by 7 (26%) mid-career researchers; 2 (7%) early career researchers and 1 (4%) participant who was not on an academic pathway. Participants were affiliated to one or more HEIs in England (19, 70%), Scotland (6, 22%) and Wales (2, 7%). Six (22%) participants were also affiliated to NHS Trusts or industry, international research organisations or not for profit organisations. Participants most often applied to NIHR (22, 81%), Medical Research Council (19, 70%) and Wellcome (11, 41%) for research funding.

### Overarching themes

**Fig 1:**
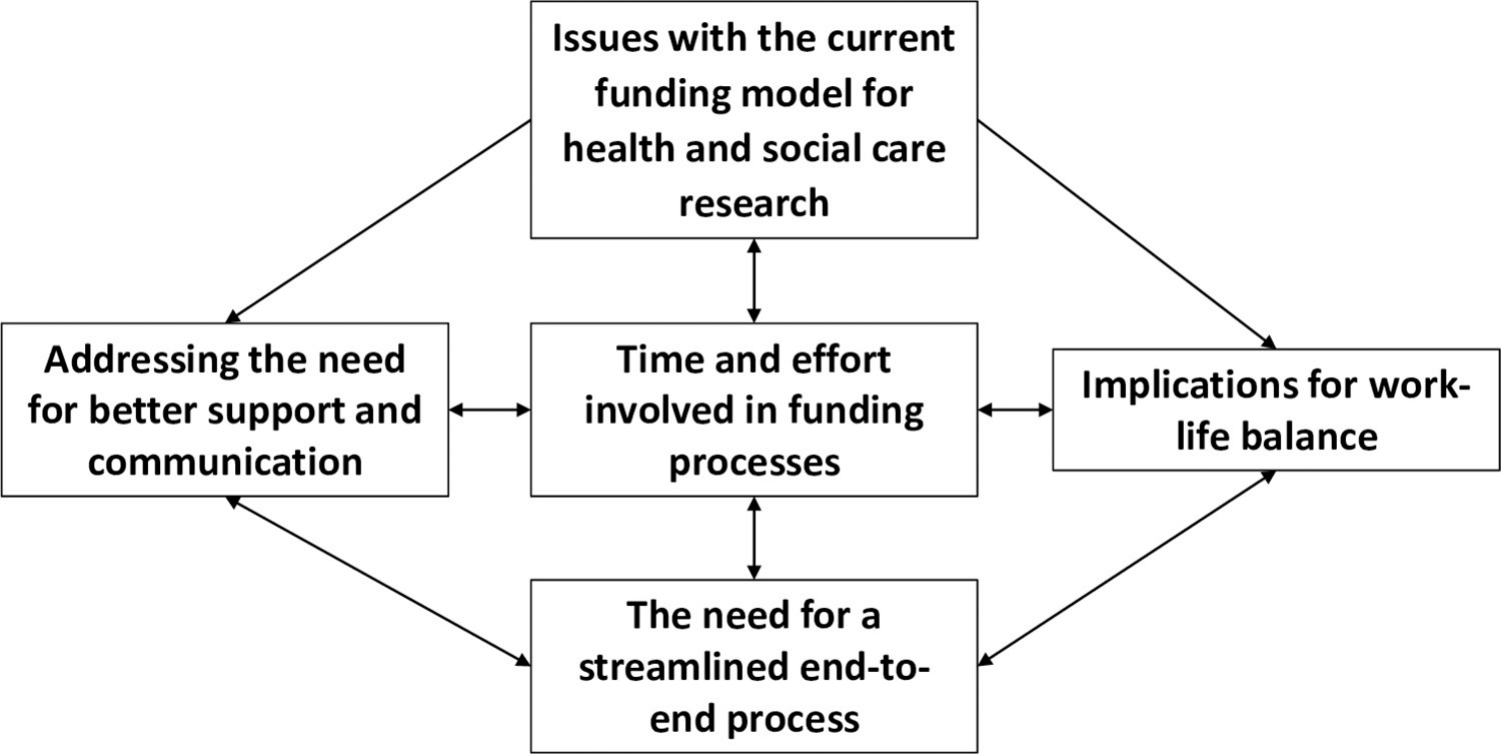
Five linked themes on researcher experiences of effort and burden associated with research funding in the UK. Arrows show directional links between the themes.

Thematic analysis identified five themes on researcher experiences of effort and burden associated with research funding in the UK (see Fig 1). As illustrated in Fig 1, the five themes are linked and the arrows between the themes show how they are interconnected. A summary of themes and associated quotes are reported in the supporting information (see S2 Table). A full description of each theme is provided below.

### Issues with the current funding model for health and social care research

A common issue raised by participants was about the current funding model used to support health and social care research. The key concept was that research is consistently being under-resourced due to funder and HEI expectations on value for money as well as perceived consequences of competitive funding, research culture and reduced budgets across the research landscape. This was felt to put pressure on researchers who had to deliver high workloads with time and resource constraints.

The budgets attached to some funding calls were suggested to be prohibitive and participants felt that despite overall rises in costs, there had been no change in what was considered expensive in terms of research. Participants described funders nearly always asking for reductions in proposed budgets and HEIs requiring certain returns on investment (e.g., overhead costs, indirect costs, full economic costing), meaning that the amount of money left to conduct the research was not a true reflection of the actual cost of the research.

Participants admitted cutting costs to research projects to make them more competitive and to increase the chance for funding success.

*And*, *actually all the incentives as I said at the beginning are to undersell the resources that are needed*, *because otherwise you look uncompetitive*. P27

However, it was questioned whether high quality research can be delivered on a low budget, especially when also needing to balance seeking new funding. Furthermore, it was felt that requests to reduce research costs at the application stage was counterintuitive as it was perceived that most projects ended up requesting extensions.

*Because*, *you know*, *with clinical trials you almost always need extensions for some reason*, *[…]*. *And they always cut your budget*, *[…] so you cut it so you can get the money and then you say*, *“Actually we need more money” and you get an extension*. P7

Participants reported feeling pressure to continually secure research funding to support their salary and other staff salaries, and due to low success rates, participants often felt frustrated that time spent on applications was wasted effort.

*A good acceptance rate is like one in five or something, so that means that 80% of the time that even good researchers spend writing proposals is essentially wasted time that doesn’t lead to anything. P11*

This is further compounded as many research activities involved in the development of an application (e.g., engaging patient and public involvement) and reporting requirements (e.g. Researchfish) are often completed outside of the funding period. The cost of these activities is not recuperated from the funder and institutions often do not provide (adequate) time for this.

*Well I think writing grants does take an enormous amount of time and it’s kind of completely sort of unfunded in many respects because you can*’*t get any funding for writing a grant*. P7

Participants also commented on the number of research staff on short, fixed term contracts who have no real job security and often leave one research project before completion to move to another project with a longer contract. Participants reflected that this could impact the delivery of a project and often the lead applicant (and other senior members of the team) are left to complete the project.

*[…] everybody is on fixed-term contracts*, *so my team could go from ten people to two or to just me*. *P1*

### Time and effort involved in funding processes

This theme relates to the overall time and effort associated with applications and reporting of research, and the impacts of experience and interactions with others. There was strong agreement that applying for research funding is time consuming, that *“the whole thing takes longer than you anticipate” (P24)*, and participants described *“a cost in terms of time that could’ve been spent doing something else” (P11*).

It is important to note that in many cases participants were not suggesting that all application or reporting processes were unnecessary, but instead were reflecting that they involved significant resource. For example, there was time and effort associated with identifying funding opportunities, developing the research questions, checking whether a research idea was *“the right application for the right funder at the right time*?*” (P9)* and then developing the costings. Participants reported a need to understand the timeframes involved in applying for funding, in particular, an awareness of how long each element of the application process would take, including those to complete before submission. For example, informing HEIs’ finance teams of intention to submit, provide sufficient time to calculate costings and ensure these were right as it was not easy to change budgets post hoc.

Participants also reported needing approvals and sign-off by various stakeholders in the researchers organisation both for an intention to apply and before the application was submitted to the funder. This was, for example, to ensure that others in the organisation are not applying for the same research funding and to check if infrastructure support is available. All of these stakeholders have their own internal timeframes which need to be factored in ahead of the funder submission deadline.

*Well*, *we need financial approval*. *We need sponsor approval*, *and we need head of department approval*, *I think that’s fairly standard*. *P20*

Participants reflected that time and stress was further exacerbated by reliance on others to complete tasks in the research pathway. They understood that people involved in approvals are often busy and under resourced, however, being *“totally beholden to the timelines of other people and departments […]*, *that’s the stress” (P1)*, and as a result could add delays to the process even when the time required for approvals has been considered.

It was suggested that applications could take on average 3–6 months to prepare. Participants appreciated funders who gave advance notice of upcoming calls or regular funding calls (e.g., bi-annually) and who provided maximum budget amounts so that they had more time to prepare and pitch their applications appropriately. Participants reflected that calls with short timeframes were not always feasible due to the difficulty and stress in getting all the costings, approvals and collaborators in place. This is further complicated when calls with short timeframes are announced near the Christmas break as institutions often close early for the holiday period, meaning internal deadlines squeeze the time available to develop the application.

*With something that’s a January deadline*: *most universities have a Christmas closure and so really you’ve got to have it ready by the middle of December*. *P1*

Short deadlines for funding calls or rebuttals were thought to be especially challenging for those who work part-time, and it was suggested that women may be more disproportionately affected by this.

*I work part time so you’re not actually giving me seven days you’re giving me less than that and if other people who work with me work part time or are clinicians who are working part time it’s impossible*. *P17*

To reduce complexity, participants indicated a preference to work with people that they had worked with before because, “*[…] you can do that quite quickly*, *whereas if it involves new expertise*, *new collaborators and new institutions then it’s a bit more complex and a bit more time consuming” (P20)*. Furthermore, patient and public involvement was something that needed to be done early in the application process and to get a representative group and for involvement to be meaningful, and not tokenistic, took time.

Another process that participants reflected on was the difficulties in addressing reviewer feedback. Whilst they were pleased to have the opportunity to respond to reviewers, the number of reviews and the length of time they had to respond sometimes made this challenging, especially when there were also contradictions between reviewer comments.

*I think the thing that can be frustrating is when you receive your reviews and you have two weeks to prepare a response*, *and then you’ve prepared your response and you’re just about to submit*, *and you get another review because there’s one that’s late*. *P21*

Participants reflected that time spent on completing reporting requirements was more proportionate than for applications, *“Compared to the time you allocate to developing the grant and submitting it*, *monitoring is not a big part of our time allocation” (P22)*. Participants indicated that the effort associated with reporting depended on the funder, institution and/or funding stream, the number of research projects that required reports, and the number of reports required after completion of the project/funding. Multiple participants mentioned the limitation of long reports or monographs, pointing out that many funders may not have the resources to manage such a process, that not everyone will end up reading these reports, and that parts of the reports will already be published in peer reviewed journals. One interviewee felt that *“bitesize papers are probably more successful” (P18)*.

Overall, there was agreement that activities involved in study set up (such as ethics, contracting, or creating advisory groups) could be challenging and time-consuming, often requiring longer timeframes to complete. The order in which to complete these tasks was discussed, with many happening in parallel.

*I find the study set up quite torturous*. *This is another bit of the whole process that takes a lot longer than most people think it will*. *P20*

There was also recognition that those with more research experience often had a better sense of timelines, costings, and where to focus effort, and were better able to tell when to pursue funding or not. It was suggested that it takes time to assimilate knowledge on funders, internal systems, writing applications and completing reporting requirements, and that this was learnt through experience.

*I mean I think the more you do the better you get at it*. *And you obviously learn through doing it*. *I’m sure you get better at it through realising what you did well in the ones that succeeded and what you didn’t do well in the ones that failed*. *And you get used to different systems for different Funders*. *P15*

However, the funding programmes/schemes often changed and it was felt that navigating the changes took time, both for the researcher and those administrative staff supporting them.

*[…] our Finance people find that the different rules for different funders which then change are difficult to manage and that generates a lot of work for them*. *P7*

### The need for a streamlined end-to-end process

Linked to time and effort involved in funding processes, participants described ineffective and inefficient processes that added time and work, and identified areas in which the end-to-end process could be streamlined. Overall, participants reflected the need for shorter research applications, although one interviewee commented that *“I do think the application forms have improved over time and have become a bit shorter and a bit more streamlined*. . .*” (P20)*. However, it was clear that others felt that this could be improved further. In particular, it was suggested that the focus of the application should be on the science and design of the research, and that the amount of additional information requested should be reduced.

*That stuff around it [the science] is the bit that I find most time-consuming*. *Well*, *that’s not true […]*. *I guess inefficient*, *like it takes up some time and from my perspective has little value*, *whereas the science takes up loads of time and has lots of value*. *P8*

It was recognised that funders often used two-stage applications to try and reduce workload for researchers and reviewers; however, it was clear that this did not translate in practice and in reality, many aspects of the research needed to be considered and approvals needed to be completed at an early stage. For two-stage application processes, it was felt that effort was front-loaded, with participants reporting having to put in as much effort at stage 1 as stage 2, despite these applications requesting less detail.

*The outline applications* [Stage 1] *are almost the full ones* [Stage 2] *with just a few pages short*. *And I often find they are almost just as much work as the full one*. *P20*

Overall, it was felt that the peer review processes employed by funders are necessary and robust. However, participants indicated short deadlines, potential biases (e.g., the appropriate reviewer for the research), and inconsistencies between different review stages that add time to information requests or affect decisions on funding success.

*Often the time between notification of success and the stage two application deadline is so short that you couldn’t start from scratch*, *you pretty much have to have the stage two application in the back of your mind or at least*, *you know*, *almost written to be ready to hit it*. *P14*

There was a sense that unless there was *“clear blue water between the outline application and the full application*, *in terms of the amount of detail required” (P25)*, that a disproportionate amount of effort and waste was seen at stage 1. In addition, some participants suggested that it would make more sense to provide certain information once the funder has expressed interest in or committed to funding a project.

*…whatever work you put in needs to be directly proportionate to the chances of getting funded*. *The stage ones*, *it should be little work*, *because it is odds-on you are going to get rejected*. *Once you get to stage two…a bit more work is required and worth a bit at that point*. *And if they have already offered you the money and they are asking you to do things*, *it is definitely worth it then because they have offered you the money*. *P8*

Participants also reported examples of repetition across different sections and stages of applications. For example, *“the forms will often have three or four boxes that seem to ask the same thing” (P17)*. Moreover, it was felt that all the different sections on the application form encourages inconsistency as it requires researchers to remember to update all the overlapping sections.

Participants felt that it was easy for funders to add requests for information to applications and reports, but that nothing seemed to be removed or checked for relevance or added value.

*Like the data management plan […] I was just particularly frustrated by the lack of relevance*, *as far as I could see*, *of it*. *(P7)*.

Similarly, more experienced participants felt that having to consult specialist services or justify their team or costings was unnecessary, “*because we have that expertise in the team” (P1)* or have a proven track record. Participants were also frustrated that templates are in general set up for randomised controlled trials, making it more difficult to complete applications and reports for different research designs.

Participants were of the opinion that funders could do more to reject applications sooner, preferably at stage 1. Having to wait months for a rejection was found to be especially frustrating if the decision came with no feedback.

*I hadn’t thought about the timing but it would be really helpful if it’s a definite no*, *so for example my colleague has just been rejected*, *her project wasn’t even sent out to peer review and she waited four months to find that out*. *P17*

Participants felt that having to update on progress too frequently via reports or oversight meetings was time-consuming and resulted in repetition of information and frustration for researchers. There was a sense from participants that annual reporting or biannual reporting is more achievable and would potentially improve quality of information and have more value to funders than more frequent (e.g., quarterly) progress updates.

*Personally I find that at least for the work that we do 12 months is a little too frequent just because things don’t change that quickly […] and actually I think you get a better quality of discussion if there’s something new to feedback and comment on*. *P2*

It was suggested that moving to real-time reporting of outputs and data would reduce the lag between the completion of research and reporting of results and would be *“something that actually shows real progress in a real time kind of way” (P26)*. Another alternative was to adjust the reporting frequency as the project progresses, as researchers may need more support and oversight at the beginning of a project. The need to keep reporting on a study long after it has been completed was also mentioned as time-consuming and potentially wasteful if not much has happened.

Whilst it was clear that participants understood the importance of monitoring and reporting, there was a desire to *“not have to report things everywhere to everyone” (P1)*. Participants felt that with many organisations requesting a lot of similar information, the online systems used to collate this information should be interoperable, allowing for the information to be easily shared across different systems.

..*and what would be really nice is if there was some*, *heaven forbid*, *linkage in a system where we’ve just submitted this to X*, *can you use this*, *rather than having to reinvent the wheel or to rewrite it all again in a different format*. *P10*

Moreover, participants reflected that online platforms were often cumbersome and not user-friendly, *“It’s a totally unusable system*. *It has been designed by somebody who does not have the end user in mind*. *It’s huge*, *it’s not intuitive; […]” (P1)*. Three main issues were raised for application platforms: formatting, registration and sign-off. Participants commented that in some online platforms character limits differed to the MS Word document they were drafted in, and formatting (bold, subheadings etc) also did not transfer, which meant re-writing already carefully written prose. Participants also commented that it could be difficult for co-applicants to register, especially non-academics such as clinicians, public or patients, and lead applicants often had to spend time assisting co-applicants with this. Similarly, some systems required sign off in a particular order or by every co-applicants which takes up time that could be used more productively.

### Implications for work-life balance

This theme captured some positive but mostly negative effects that the current research environment had on researchers. Overall, participants felt that the UK research environment (all participants were affiliated with UK organisations) fostered imbalances in wellbeing and work-life activities. Participants reflected that many research activities are completed outside of normal working hours and they described the processes of writing an application and reporting as pressurised and emotionally draining, causing stress, anxiety and reduced morale.

*I think we are all under a fair amount of pressure and we’re all used to it*, *[…] I accept that if I’ve got a deadline in ten days for a straight to stage two*, *I’ll be working on that every evening after I put the kids to bed and around when I’ve got them […] you know*, *to try and hit deadlines and get stuff done*. *We were emailing at 11*.*30 on Friday night*. *It’s a lot of pressure*. *P23*

Participants indicated that this was felt to be especially pronounced for lead applicants, who often had disproportionate workloads compared to other co-applicants and early career researchers who had more at stake than senior colleagues as “*[…] a failed grant might hold their career back for a few more years” (P8)*.

*Then somebody in the group has to decide that they are willing to be the PI of that project*, *so an awful lot of funding ideas just disappear because everybody is happy to be part of a project but nobody is willing to lead it*. *I think the burdens of leading a project are massive*! *P27*

Indeed, perceived inequalities in career stage, affiliation, and gender for example, were seen as unfair and impacting on chances of success in research.

There was also a perception that some lead applicants spend more than their allotted time percentage on a research project. This not only left them feeling like an administrator or manager rather than a researcher, but participants commented that it resulted in a trojan horse effect, whereby contributions of all applicants may not be accurately represented.

*The lead applicant goes in around 10% and the co-apps at around 5%*. *And it doesn’t feel like the co-apps do half the amount of work that the main app does*, *you know*. *P23*

Participants reflected that writing applications was often conducted alongside their day jobs (e.g. clinicians, lecturers) and so there was a constant juggle of tasks. Although completing applications and reports was considered “*part and parcel of the role” (P4)*, it was felt that institutions did not give adequate time or support to do this, and the time and effort required to complete these was not always acknowledged or recognised, especially when allocating workload or when applications were unsuccessful.

Overall, participants’ despondence with the research environment in the UK was palpable. They described how low success rates, strong competition between and within institutions, constant rejection and limited incentives made it difficult to persevere with a research career. It was felt that many talented researchers are being lost post-training to better paid and more reliable careers, for example the pharmaceutical industry. Although research organisations (e.g. HEIs and NHS trusts) were seen as supportive of research, it was suggested that the UK needs to invest more in talented early career researchers and provide a more attractive and enabling research environment.

### Addressing the need for better support and communication

Participants felt that organisational support and communication was received from HEIs, NHS trusts, funders and other services such as the research design service or clinical trials units. However, whilst participants felt that having a central administrative or professional service support from HEIs was invaluable, it was clear that the level of support available was dependent on the institution, department, and career stage or success. Furthermore, it was suggested that central administrative or professional services themselves tended to be under-resourced which led to inconsistency in support and effects on timelines.

*There’s no consistency of people*, *and it just feels like you send emails and they go into a black hole sometimes*. *P4*

Most participants required, and received, support for costings and financial reconciliations, as they felt they did not have the right expertise for this (especially for Schedule of Event Cost Attribution Template (SoECAT) forms). They also reported needing support with understanding requirements from certain funders or organisational policies, project management and reporting requirements. For example, some participants described how they were unaware of the reporting requirements of a funder until they were asked to report or unless they had been successful previously.

Some participants indicated that application and reporting guidelines provided by funding organisations were clear and helpful, however others felt uncertain about the content, detail and structure required for different sections of applications or reports.

*I think for some of the headings in an application form*, *I think a little bit more information about what it is you’re meant to be putting into*, *you know*, *what type of content is needed*, *because sometimes the language is actually quite hard*, *you know*, *when you read it and you think*, *“I think I know what this means*,*” and then you start writing and you think*, *“No*, *I don’t*.*” P16*

Similarly, some participants indicated that more clarity was needed on funding organisation priorities, expectations for research applications, and how these affect decision making for funding allocation..

*As I said*, *from the funder*, *I think clearer guidelines about expectations and how things are shortlisted*. *Because that’s where it’s a bit of a black box at the moment*, *for us*. *Which is why we put in so much work to prepare for them*. *P5*

Participants reported that it was helpful to have access to previously submitted applications to get a sense of what to write and how to pitch ideas. This was suggested to be especially helpful for more junior applicants or when applying to a new funder. It was acknowledged that colleagues and collaborators tended to be generous in letting others read successful applications, although it was noted that some researchers were more cautious about sharing unsuccessful applications.

A good peer support network was regarded as invaluable, with one interviewee commenting, *“the biggest support is peer-to-peer support” (P15)*. Having senior colleagues to act as mentors and advocates was suggested to be especially important for early career researchers

*Yeah*, *so no*, *so you know*, *when you’ve got a great colleague*, *you receive a lot of wonderful support*, *you know*, *work collaboratively*, *and that’s really good*, *and the other tension is when somebody doesn’t*, *there’s a mismatch in expectations*. *P16*

With respect to reporting, some participants were unsure how information in reports is used. This was compounded by the fact they felt that they were not given meaningful feedback following the submission of a report, so they did not know if it had been read or whether the content was what was expected or valued. Participants indicated that they might tailor reports or spend more time on reports if they knew the purpose for the information being requested. It was acknowledged, however, that some funders were better than others at communicating the purpose of the report and in providing feedback.

*But I think the challenge is it’s not always clear how this information is being used*, *whether it’s being used*, *which goes back to that point about can we do this in a way that allows people to feel like the time that they’re spending on this stuff is actually well spent*. *P21*

The importance of constructive feedback was also recognised as a way to improve applications and the overall quality of funded research, “*If you want better applications you have to provide feedback*, *there is no way around it” (P14)*. Participants commented that, although it took time, it was valued to have an opportunity to clarify detail or queries in rebuttals; however, this was only considered helpful if the application has a real chance of being funded.

*Feedback is really critical to working out what to do next time*, *whether that’s revamping the same grants*, *whether it’s ditching it and doing something completely different*. *P15*

Participants reported that communication with funders was seen as responsive and helpful, especially responses to email queries. Furthermore, it was recognised that good communication is two-way and that it was important to notify funders about changes to funded research. However, it was felt that funders do not always encourage onward dialogue and that some interactions would be more beneficial if they were verbal discussions. For example, for advice on remit and programme selection; for further clarification on feedback; to discuss changes that need to be made and how this might affect delivery of the project; and when reporting, both to update progress and get clarifications.

Participants also voiced the need for more understanding from the funder about the tendency for research timelines and goals to drift, and to be more flexible with these. For example, by acknowledging that unforeseen circumstances and delays in the funding process could alter research milestones, but not affect the overall quality of the research.

*…the milestones are the ones on the Gantt chart and comparing yourself to the wishful thinking you had at grant application stage to the reality that you face during the study is a soul-consuming process really. It’s very hard. P12*

## Discussion

This study has provided insight and understanding into what researchers in the field of health and social care perceive as effortful and burdensome in the end-to-end research process, from application through post award management including study set up, data collection and reporting. Thematic analysis of interview data identified key areas in the research lifecycle that consume researchers time and effort, and identified areas that were felt could improve researcher experiences in the more immediate and longer term. For example, streamlining and open communication. A summary table of considerations for funding organisations, HEIs or others (e.g. other research organisations, regulators or researchers themselves) are provided in the supporting information (see S3 Table).

In line with others, the findings from this study highlight the complexity between the researcher, HEI and funder and shows that there is considerable researcher effort and burden in the application, delivery and reporting stages of research. Whilst it was clear that some of these activities are necessary–for instance, the time needed to develop a high-quality application and conduct research–it was also clear that inefficient and ineffective processes are producing and contributing to unnecessary researcher burden, research waste and negatively affecting research culture (the expected behaviours, values and attitudes of research communities).

The findings demonstrate that at the heart of many of the issues faced by researchers is that of lack of financial resource for research and the research culture (e.g. expected behaviours) that this generates. Value for money is an important consideration for funders, is included on their assessment criteria for funding research and is often raised as an issue in feedback to applicants (e.g., [22–24]). This study further highlighted that value for money is a high priority for institutions too, with high overhead charges and need for returns on investment increasing research costs or ruling out funding organisations who do not pay full economic costings. This was described as prohibitive to some researchers applying, and ultimately they have to make the decision as to whether they should cut costs and knowingly under-resource their projects to make them appear viable and competitive or to not apply. Both of these options may have significant implications for researchers both in terms of work-life balance and career progression. The process of reducing costs at the application stage also appears counterproductive as findings indicated a perception that these costs were nearly always requested back further down the line of a successful application. Whilst this claim needs further research to substantiate it, if true it raises the question of how much time and effort is being used/wasted by researchers and funders in these negotiations.

Lack of, or under resourcing of, true research costs were shown to have knock on effects to the delivery and quality of the research (see also [13, 18]). For example, reducing the number of researchers employed on the research or lack of job security for researchers may result in too few staff available to conduct and deliver the research. Junior members of staff do not get the valuable experience of finishing and writing up the research and the lead applicant has to take on more responsibilities which have to be completed alongside other activities such as teaching, mentoring and clinical roles. Consequently this exacerbates already stretched workloads and impacts on work-life balance and well-being, as many research activities are conducted outside of normal working hours [10, 11, 13].

Taken together, these issues indicate a need for a better understanding of what value for money in research really looks like in today’s society, and what the true costs of a research study are, including pre-application (e.g., preliminary data collection) and reporting activities that are completed outside of the funding window. This is in line with the recent Independent Review of the UK’s Research, Development and Innovation Organisational Landscape [18] in which it was recommended that proper ‘end to end’ funding is required to fully support research activities. Similarly, the R&D People and Culture strategy [25] emphasises the need to better understand the impacts of the approach to research funding as well as to invest in better incentives to attract and retain researchers in the UK.

The need for fewer but better proposals was raised and it was suggested that funders should consider more carefully the quality of research they fund (i.e., ensure the research is methodologically sound), as money invested into ‘bad’ research stops high quality work from being funded, which ultimately leads to research waste [16, 26]. For example, Pirosca et al [26] estimated that high bias due to poor research design accounted for research waste of 62% of randomised clinical trials (from a sample of 1659). Accountability and best use of public spending is especially important in the current cost of living crisis in the UK, and in light of governmental initiatives to reduce research bureaucracy and foster excellence in the UK research environment [14, 15, 18].

As described in the R&D People and Culture Strategy [25], it is important to have the right people with the right skills in post. In line with this, one way to facilitate increasing the quality of funded research would be for funders to invest in strengthening methodological expertise on funding committees via training or recruitment of methodologists [26]. Another option could be for funders to collaborate with research teams to help them revise research applications that they are interested in funding but need additional work.

Inefficient processes further add to research waste and the frustrations that many researchers experience. The need for transparency and streamlining are not new or surprising findings, with many studies and reviews describing them as key concepts to be improved [15, 18, 22–24, 27] and ways in which they can be improved [3, 6, 24, 28–30]. For example, Fackrell et al [23] described the need for transparency and clarity in feedback comments and decision-making as the most reported theme exploring researchers’ perceptions of funding committee feedback. These studies demonstrate there is a need for more explicit expectations and guidelines for application and reporting content, as well as more constructive feedback and justification of decisions and requests for information. Indeed, the importance of closing the feedback loop so that researchers better understand why information is requested, whether they provided the right information and how the information is being used was described as critical in facilitating researchers to improve the quality of the information they provide [3, 6, 27].

There is also a perception that many processes in the research pipeline have lost focus of the science they are there to promote, and that the research sector has entered a self-perpetuating state of information requests that are continually added too, with no review of relevance or value [31]. For example, despite there being no substantial change in the law for clinical trials since 2001, a perceived significant increase in administrative requirements for clinical research has been reported [2]. Furthermore, a shift in administrative effort and burden within HEIs has also been reported with automated reporting and compliance processes passed to the researcher to complete [31]. Similar to other work [32], administration relating to the financial management of research was reported to be particularly high due to the iteration and negotiation that encompassed costs and lack of researcher expertise in this area. Guidance and support in this area were welcomed by most participants, in particular, the need and benefit of institutional research and administrative support. However, support was again perceived to be undermined by a lack of investment in centralised teams, which resulted in limited or inconsistent support that differed across institutions and departments.

A strength of this study is that the findings are interpretations from a dataset of rich, in-depth interviews with researchers who have experience of applications and reporting from a broad range of UK health and social care funding organisations and affiliated research organisations. However, a limitation is that as the sample includes views of more senior, white and/or British researchers, they may be open to bias. For example, senior researchers may have different views and experiences to those earlier in their career. The high proportion of senior researchers and white and/or British researchers in our sample is in line with other studies (e.g., [1]). Although this may be due to our sampling technique, it is likely this reflects the wider issue of diversity in health and social care research. This issue has been recognised and the R&D People and Culture strategy [25] highlighted narrative CVs as an initiative to increase diversity and inclusion in research. As such, many research organisations, including UK and international research funders, have implemented or are piloting the use of narrative CVs in the assessment of research funding and promotion [33, 34]. Furthermore, as our findings were consistent across our participants, we think it unlikely that key themes would have been extracted with a more diverse sample; however, future work should explore this further to explicitly compare experiences and opinions across different participant groups.

In conclusion, this study highlighted that there are many areas along the research pathway that are associated with tasks that take considerable time and effort, some of which are perceived as unnecessary. Whilst researchers are not adverse to dedicating significant proportions of their time to these tasks, this time and effort could be put to better use and encourage a more enabling research environment. Better investment in research and the researchers who design and complete these studies and improvements in transparency, streamlining and support could significantly reduce perceived unnecessary researcher burden. Although many considerations to improve researcher experiences fall to the funder to instigate, collaborative efforts between all stakeholders (research funders, HEIs, research organisations, regulators and researchers) are required to ensure a more positive research culture can flourish, ultimately improving the quality of research that is funded leading to better outcomes for patients and public.

## Supporting information

S1 Table Interview topic guide. (DOCX)

S2 Table Table of themes, key concepts and quotes of researchers experiences of UK funding processes. (DOCX)

S3 Table Summary table of considerations for reducing researcher burden and research waste from researchers experiences. Considerations are derived directly from interview data of researcher experiences. HEI = higher education institution; other refers to other research organisations, researchers or wider research community.
(DOCX)

## References

[1] University and College Union. Workload survey 2021: Data report [Internet]. 2022 [cited 2023 Mar 22]. Available from: https://www.ucu.org.uk/media/12905/UCU-workload-survey-2021-data-report/pdf/WorkloadReportJune22.pdf.

[2] Rule S, LeGouill S. Bureaucracy is strangling clinical research. BMJ. 2019; 364: l1097. doi: 10.1136/bmj.l1097 30867145

[3] Rodriguez Rincon D, Feijao C, Stevenson C, Evans H, Sinclair A, Thomson S, et al. Study on the proposal evaluation system for the EU R&I framework programme: Final report. [Internet]. RAND Europe. Publications Office of the European Union; 2022 [cited 2023 Mar 22]. p. 53. Available from: https://data.europa.eu/doi/ doi: 10.2777/16211

[4] Graves N, Barnett AG, Clarke P. Funding grant proposals for scientific research: Retrospective analysis of scores by members of grant review panel. BMJ. 2011; 343: d4797. doi: 10.1136/bmj.d4797 21951756 PMC3181233

[5] Barnett AG, Herbert DL, Campbell M, Daly N, Roberts JA, Mudge A, et al. Streamlined research funding using short proposals and accelerated peer review: an observational study. BMC Health Serv Res. 2015; 15: 55. doi: 10.1186/s12913-015-0721-7 25888975 PMC4324047

[6] Guthrie S, Ghiga I, Wooding S. What do we know about grant peer review in the health sciences? [version 2; referees: 2 approved]. F1000 [Internet]. 2018; 6: 1335. 10.12688/f1000research.11917.1 PMC5883382 29707193

[7] Von Hippel T, Von Hippel C. To apply or not to apply: A survey analysis of grant writing costs and benefits. PLoS One. 2015; 10: e0118494. doi: 10.1371/journal.pone.0118494 25738742 PMC4349454

[8] Herbert DL, Barnett AG, Clarke P, Graves N. On the time spent preparing grant proposals: An observational study of Australian researchers. BMJ Open. 2013; 3: e002800. doi: 10.1136/bmjopen-2013-002800 23793700 PMC3664356

[9] Baker S. UKRI success rates fall as grant applications ramp up. Times Higher Education [Internet]. 2021 Jul 28 [cited 2023 Mar 22]; Available from: https://www.timeshighereducation.com/news/ukri-success-rates-fall-grant-applications-ramp.

[10] Christian K, Johnstone C, Larkins JA, Wright W, Doran MR. A survey of early-career researchers in Australia. Elife. 2021; 10: 1–19. 10.7554/eLife.60613 PMC7800379 33423739

[11] Nicholls H, Nicholls M, Tekin S, Lamb D, Billings J. The impact of working in academia on researchers’ mental health and well-being: A systematic review and qualitative metasynthesis. PLoS One. 2022May 1. 17: e0268890. 10.1371/journal.pone.0268890 PMC9132292 35613147

[12] Bartlett MJ, Arslan FN, Bankston A, Sarabipour S. Ten simple rules to improve academic work- life balance. PLoS Comput Biol. 2021 Jul 1; 17: e1009124. doi: 10.1371/journal.pcbi.1009124 34264932 PMC8282063

[13] Wellcome. What Researchers Think About the Culture They Work In [Internet]. 2020 [cited 2023 Mar 22]. Available from: https://wellcome.org/sites/default/files/what-researchers-think-about-the-culture-they-work-in.pdf.

[14] Department for Science Innovation and Technology, Department for Business Energy and Industrial Strategy. UK Research and Development Roadmap [Internet]. 2020 [cited 2023 Mar 22]. Available from: https://assets.publishing.service.gov.uk/government/uploads/system/uploads/attachment_data/file/896799/UK_Research_and_Development_Roadmap.pdf.

[15] Department for Science Innovation and Technology, UK Research and Innovation, Department for Business Energy & Industrial Strategy. Independent Review of Research Bureaucracy Final Report [Internet]. 2022 [cited 2023 Mar 22]. Available from: https://assets.publishing.service.gov.uk/government/uploads/system/uploads/attachment_data/file/1094648/independent-review-research-bureaucracy-final-report.pdf.

[16] Glasziou P, Chalmers I. Research waste is still a scandal-an essay by Paul Glasziou and Iain Chalmers. BMJ. 2018; 363: k4645.

[17] Glasziou PP, Sanders S, Hoffmann T. Waste in covid-19 research. BMJ. 2020; 369: m1847. doi: 10.1136/bmj.m1847 32398241

[18] Department for Science Innovation and Technology, Department for Business Energy and Industrial Strategy. Independent Review of the UK’s Research, Development and Innovation Organisational Landscape: Final Report and Recommendations [Internet]. 2023 [cited 2023 Jul 13]. Available from: https://assets.publishing.service.gov.uk/government/uploads/system/uploads/attachment_data/file/1141484/rdi-landscape-review.pdf.

[19] Fackrell K, Church H, Crane K, Recio-saucedo A, Blatch Jones A, Meadmore K. Exploring researcher experiences of UK research funding: A survey study. In: Faculty of Medicine Research Conference [Internet]. Southampton; 2023 [cited 2023 Jul 20]. Available from: https://pure.soton.ac.uk/admin/files/133960873/Fackrell_FoM_poster.pdf.

[20] Tong A, Sainsbury P, Craig J. Consolidated criteria for reporting qualitative research (COREQ): A 32-item checklist for interviews and focus groups. International Journal for Quality in Health Care. 2007; 19: 349–57. doi: 10.1093/intqhc/mzm042 17872937

[21] Braun V, Clarke V. Thematic Analysis. A practical guide. London: SAGE; 2022.

[22] Meadmore K, Fackrell K, Recio-Saucedo A, Bull A, Fraser SDS, Blatch-Jones A. Decision-making approaches used by UK and international health funding organisations for allocating research funds: A survey of current practice. PLoS One. 2020; 15: e0239757. doi: 10.1371/journal.pone.0239757 33151954 PMC7644005

[23] Fackrell K, Meadmore K, Recio-Saucedo A, Bull A, Fraser S, Blatch-Jones A. Identification and comparison of key criteria of feedback of funding decisions: Mixed-methods analysis of funder and applicant perspectives. BMJ Open. 2021 Sep 17; 11: e048979. doi: 10.1136/bmjopen-2021-048979 34535478 PMC8451298

[24] Coveney J, Herbert DL, Hill K, Mow KE, Graves N, Barnett A. ‘Are you siding with a personality or the grant proposal?’: observations on how peer review panels function. Res Integr Peer Rev. 2017; 2: 19. doi: 10.1186/s41073-017-0043-x 29451548 PMC5803633

[25] Department for Science Innovation and Technology, Department for Business Energy and Industrial Strategy. R&D People and Culture Strategy People at the heart of R&D [Internet]. 2021 [cited 2023 Jul 13]. Available from: https://assets.publishing.service.gov.uk/government/uploads/system/uploads/attachment_data/file/1004685/r_d-people-culture-strategy.pdf.

[26] Pirosca S, Shiely F, Clarke M, Treweek S. Tolerating bad health research: the continuing scandal. Trials. 2022; 23: 458. doi: 10.1186/s13063-022-06415-5 35655288 PMC9161194

[27] Gluckman P, Ferguson M, Glover A, Grant J, Groves T, Lauer M, et al. International Peer Review Expert Panel: A report to the Governing Council of the Canadian Institutes of Health Research [Internet]. 2017. Available from: http://www.cihr-irsc.gc.ca/e/50248.html.

[28] Recio-Saucedo A, Crane K, Meadmore K, Fackrell K, Church H, Fraser S, et al. What works for peer review and decision-making in research funding: a realist synthesis. Res Integr Peer Rev. 2022; 7: 2. doi: 10.1186/s41073-022-00120-2 35246264 PMC8894828

[29] Herbert DL, Graves N, Clarke P, Barnett AG. Using simplified peer review processes to fund research: a prospective study. BMJ Open. 2015; 5: e008380. doi: 10.1136/bmjopen-2015-008380 26137884 PMC4499682

[30] Guthrie S. Innovating in the research funding process: Peer review alternatives and adaptions [Internet]. 2019. Available from: www.academyhealth.org/ParadigmProject.

[31] Bozeman B, Youtie J. Robotic Bureaucracy: Administrative Burden and Red Tape in University Research. Public Adm Rev. 2020; 80: 157–62.

[32] Arvizu DE, Droegemeier KK, Córdova FA, David R, Gulari E, Lepage GP, et al. National Science Board. Reducing investigators’ administrative workload for federally funded research [Internet]. 2014 [cited 2023 Mar 22]. Available from: https://www.nsf.gov/pubs/2014/nsb1418/nsb1418.pdf.

[33] Meadmore K, Recio-Saucedo A, Blatch-Jones A, Church H, Cross A, Fackrell K, et al. Exploring the use of narrative CVs in the NIHR: a mixed method qualitative study [Internet]. 2022 [cited 2023 Jul 13]. Available from: https://openresearch.nihr.ac.uk/documents/2-38.

[34] UK Research and Innovation. Résumé Resources Library: support for adopting narrative CVs [Internet]. 2023 [cited 2023 Jul 20]. Available from: https://www.ukri.org/what-we-do/supporting-healthy-research-and-innovation-culture/research-and-innovation-culture/supporting-the-community-adoption-of-r4r-like-narrative-cvs/resume-resources-library-support-for-adopting-narrative-cvs/.

