# Supplementary material for: An in-depth exploration of researcher experiences of time and effort involved in health and social care research funding in the UK: The need for changes
Source: PLoS One. 2023 Sep 21;18(9):e0291663. doi: 10.1371/journal.pone.0291663 (PMC10513312; doi:10.1371/journal.pone.0291663)
Supplement: S1 Table — (DOCX) [file pone.0291663.s001.docx]

**S1 Table. Interview topic guide**

| **Application processes** | |
| --- | --- |
| Describe experience and challenges | **Thinking about time and effort, can you talk me through the process of applying for funding?** |
|  | *What processes or approvals did your institution require you complete? (e.g., finance, ethics/sponsor, R&D, internal/external peer review)* |
|  | *What is your experience of the peer review process?* |
|  | **Did you have any difficulties completing these processes? If so, what difficulties?** |
|  | *How does the application process work with co-applicants?* |
|  | *What took longer than you expected to complete? If so, why did it take longer?* |
|  | **Was there any part of the process you felt was repetitive or unnecessary?** |
|  | **What information or support would have been helpful during the process?** |
| Critical information | **In your opinion, what information do you think is key for making a funding decision?** (what sections on the application form) ** [ask if stage 1 or stage 2] |
|  | *If two-stage application, which information do you think is important to provide at stage 1? (e.g., the team information, CV, plain English summary)* |
|  | *How many pages do you need to make a case for your research project?* |
|  | *What supporting information do you think is necessary to include? (e.g., flow diagram, logic model)* |
| Timeframes | **Does the timing of the funding call affect whether you will submit an application?** **  (e.g. over Christmas break)  (e.g. short timeframe for call) |
|  | **Are you given enough time to respond to funding committee feedback?** |
|  | *Once a decision has been made, would it be useful to know where you were ranked in the list of fundable projects? (e.g., if two projects were funded – you were ranked 10^th^)* |
| Support | **Do you look at previous applications when you are planning and writing an application?** |
|  | *Where do you access these?* |
| **Monitoring processes** We are now going to move to talking about monitoring processes | |
| Describe experiences and challenges | **Again thinking about time and effort, can you describe what a (typical) monitoring and reporting process involves?** |
|  | *Which organisations require reports and how often?* |
|  | *What information do organisations typically ask for (e.g. progress, recruitment or impact)?* |
|  | **Can you think of any examples where you were unsure what information is being requested and what you did?** |
|  | *Was this related to a specific organisation?* |
|  | **Were there any other challenges in completing the reporting requirements?** |
|  | *Did any of these reports take longer than you expected to complete? If so, why did it take longer?* |
|  | **Was there any part of the process you felt was repetitive or unnecessary?** |
|  | **What support would have been helpful during this process?** |
| Purpose | **How do you think the information you provide is used by different organisations?** |
|  | **Would knowing the purpose make a difference in how you feel about completing the reports and the time you allocate to the task?** |
| Critical information | **What, if any, information about your research do you think is important to report?** |
|  | *What information would you need to provide to show that your project is on track?* |
|  | *How often would you expect to have to report on your progress?* |
|  | **What changes in your research plan/grant/protocol do you think these organisations should be told?** |
|  | *What would you consider a minor vs major change?* |
| Support | *Are you required to acknowledge your funder in publications? If yes/no do you know what to write?* |
| **Burden** | |
|  | **How do you feel all these requirements and processes impact you as a researcher?** |
|  | **Do you receive the support that you need to write and submit an application and also to complete reporting requirements? (give examples of what said in previous sections)** |
|  | *What (other) support do you need from your funder/institution? (e.g., people or training)* |
|  | *What (other) support do you receive from your colleagues?* |
|  | **Are your efforts recognised by your employer/supervisor/line manager?**  (e.g. recognise the time and effort that you putting in/ taken to apply for funding/monitoring as well as doing your job (research/lecturing) or recognising leadership skill) |
|  | **Do you think that career stage makes a difference in how you experience application and reporting processes?** |
| Anything else | **Is there anything else you would like to tell us about your experiences submitting applications or completing reporting requirements?** |
