# Supplementary material for: An in-depth exploration of researcher experiences of time and effort involved in health and social care research funding in the UK: The need for changes
Source: PLoS One. 2023 Sep 21;18(9):e0291663. doi: 10.1371/journal.pone.0291663 (PMC10513312; doi:10.1371/journal.pone.0291663)
Supplement: S2 Table — (DOCX) [file pone.0291663.s002.docx]

**S2 Table.** Table of themes, key concepts and quotes of researchers experiences of UK funding processes

| **Theme**  Key concepts | **Example quotes** |
| --- | --- |
| **Issues with the current funding model for health and social care research**   - The need for researchers to continually chase/apply for research funding - Frustration with and wasted effort due to low success rates - Institute requirement for cost recovery/return on investment in research - Consequences of a lack of permanent contracts for research staff - Perceptions that research is under-resourced - Research activity that is not funded - The commonness of requests for cost and no cost extensions to studies | Well just the fact that because we have to seek money from so many different sources we’re having to look in lots of different directions for all of these things. P19  Well it’s just par for the course really, you know, we’re researchers, we’re academics, applying for funding is basically what we do. I mean, I know for some universities it’s part of the job that you have to submit a certain number of applications per year; P26  A good acceptance rate is like one in five or something, so that means that 80% of the time that even good researchers spend writing proposals is essentially wasted time that doesn’t lead to anything. P11  The only thing we [the university] are at the moment concerned about is cost recovery. P10  Oh, it’s horrendous. I don’t know why anybody comes into research if they’ve not got a proper job because they are all in fixed-term contracts and so my team is everybody is on fixed-term contracts, so my team could go from ten people to two or to just me. P1  And, actually all the incentives as I said at the beginning are to undersell the resources that are needed, because otherwise you look uncompetitive. P27  Well I think writing grants does take an enormous amount of time and it’s kind of completely sort of unfunded in many respects because you can’t get any funding for writing a grant. P7  Because, you know, with clinical trials you almost always need extensions for some reason, obviously particularly at the moment with the pandemic, but it’s very, very common just because they are quite difficult to do and obviously you’ve never done that study ever before so you hit unknown problems which slow things down. And they always cut your budget, you know, you put in how much you want and they say, “Cut it,” so you cut it so you can get the money and then you say, “Actually we need more money” and you get an extension. P7 |
| **Time and effort involved in funding processes**   - Time and effort involved in developing research applications - Time and effort involved in reporting - Awareness of timeframes for all elements of an application - The time and stress associated with reliance on others in the application or research process - The challenges of study set up - Effects of experience in the end to end research process - The value of reporting - Difficulties of addressing reviewer feedback - Navigating and ‘keeping up with’ variation in requirements and processes of different organisations | The whole thing takes longer than you anticipate. P24  So there’s definitely a cost in terms of time that could’ve been spent doing something else. P11  I almost try and allow for it to take a lot longer than I think it’s going to take and then it still takes even longer than that. A lot of that is waiting for Co-applicants to do something. P3  Compared to the time you allocate to developing the grant and submitting it, monitoring is not a big part of our time allocation. P22  I work part time so you’re not actually giving me seven days you’re giving me less than that and if other people who work with me work part time or are clinicians who are working part time it’s impossible. P17  So, I think it is all of the stuff that is not in our control and it’s stressful because it’s not I our control so we are totally beholden to the timelines of other people and departments and we have no influence on it. So, that’s the stress. P1  I find the study set up quite torturous. This is another bit of the whole process that takes a lot longer than most people think it will. P20  I mean I think the more you do the better you get at it. And you obviously learn through doing it. I’m sure you get better at it through realising what you did well in the ones that succeeded and what you didn’t do well in the ones that failed. And you get used to different systems for different Funders. P15  I don’t mind reminding myself of what I’ve done because obviously you can forget from time to time and it’s always good to remind yourself what you’ve done and log it somewhere. P6  And sometimes it can be a useful process because it forces you to reflect on where you are or to highlight things that you’re having difficulties with so actually for me monitoring has been okay. P17  I think the thing that can be frustrating is when you receive your reviews and you have two weeks to prepare a response, and then you’ve prepared your response and you're just about to submit, and you get another review because there's one that's late. […] but that's one of the things that I know just really throws people because you've squeezed all of your response into two pages and then suddenly you have to find a way to respond to another review that appeared out of nowhere. P21  […] our Finance people find that the different rules for different funders which then change are difficult to manage and that generates a lot of work for them. P7 |
| **The need for a streamlined end-to-end process**   - Front-loaded and disproportionate effort involved in applications - Repetition of information in applications and reports - Processes in applying for funding or reporting that are perceived as unnecessary or irrelevant and may not add value - Clarification of expectations of content for applications and reports - The need for shorter applications and how that can be achieved - Simpler online platforms that work across organisations - Reduce the amount of reporting that is required - Need for funder flexibility with application forms, research timelines and reporting schedules | The outline applications are almost the full ones with just a few pages short. And I often find they are almost just as much work as the full one. P20  …whatever work you put in needs to be directly proportionate to the chances of getting funded. The stage ones, it should be little work, because it is odds-on you are going to get rejected. Once you get to stage two…a bit more work is required and worth a bit at that point. And if they have already offered you the money and they are asking you to do things, it is definitely worth it then because they have offered you the money. P8  There was clear blue water between the outline application and the full application, in terms of the amount of detail required, so yeah, the second stage was very much building on the first stage. I don’t think there was any real redundancy there. P25  In terms of the repetition the forms will often have three or four boxes that seem to ask the same thing. P17  That stuff around it [the science] is the bit that I find most time-consuming. Well, that’s not true […]. I guess inefficient, like it takes up some time and from my perspective has little value, whereas the science takes up loads of time and has lots of value. P8  Like the data management plan was a particularly...I remember that one because I was just particularly frustrated by the lack of relevance, as far as I could see, of it. P7  As I said, from the funder, I think clearer guidelines about expectations and how things are shortlisted. Because that’s where it's a bit of a black box at the moment, for us. Which is why we put in so much work to prepare for them. P5  I do think the application forms have improved over time and have become a bit shorter and a bit more streamlined...P20  ..and what would be really nice is if there was some, linkage in a system where we've just submitted this to X, can you use this, rather than having to reinvent the wheel or to rewrite it all again in a different format. P10  It’s a totally unusable system. It has been designed by somebody who does not have the end user in mind. It’s huge, it’s not intuitive; […]. P1  And we don’t mind reporting things to our Funder, that’s fine, but let’s just do that in as simple straightforward way as possible; let’s not have to report things everywhere to everyone. P1  Personally I find that at least for the work that we do 12 months is a little too frequent just because things don’t change that quickly and these are busy people, so I don’t want to impose too much on their time and actually I think you get a better quality of discussion if there’s something new to feedback and comment on. P2  The feedback we got [from the funder] was that it was fine to submit subsequent progress reports at 12-month and six-month intervals, which was obviously very welcome, so I think that kind of flexibility is actually extremely valuable…rather than dealing with a system that doesn’t respond to the input, just sort of mechanically requires the same report, regardless of whether it’s actually redundant at six months to submit yet another report. P25 |
| **Implications for work-life balance**   - Recognising and acknowledging effort involved in writing applications and conducting research - Disproportionate expectations on lead applicants - Impacts of applications and reporting processes on researchers’ wellbeing and work-life balance - Acceptance by researchers that all processes are ‘part of the job’ - Perceived inequalities (e.g., seniority, gender, affiliation) that arise in applying for research funding - Research careers are becoming less attractive | I mean they are not recognised in workload allocation, I think that is the biggest battle is nobody seems to understand how much time research actually takes. P27  The lead applicant goes in around 10% and the co-apps at around 5%. And it doesn’t feel like the co-apps do half the amount of work that the main app does, you know. P23  Then somebody in the group has to decide that they are willing to be the PI of that project, so an awful lot of funding ideas just disappear because everybody is happy to be part of a project but nobody is willing to lead it. I think the burdens of leading a project are massive! P27  I think we are all under a fair amount of pressure and we’re all used to it, and that’s maybe not universally a good thing, so, you know, I accept that if I’ve got a deadline in ten days for a straight to stage two, I’ll be working on that every evening after I put the kids to bed and around when I’ve got them […] you know, to try and hit deadlines and get stuff done. We were emailing at 11.30 on Friday night. It’s a lot of pressure. P23  I kind of think it’s part and parcel of the role. I mean, if it can be streamlined, then great, but I do feel we should be called to account for, you know, we’re given quite large amounts of money and I feel we should be called to account for them, so I feel it’s kind of part of the job really. P4  And also, for early career researchers who know that they need to get grants for promotion, and because it is so competitive, and in our field particularly it feels like there is very little money, then they know that a failed grant might hold their career back for a few more years because they need a grant of some size on their CV for them to be able to progress. P8  But, I am not the only researcher I know who is just thinking that it has just all become impossible. P27 |
| **Addressing the need for better support and communication**   - Access to previous application and funded project information - Verbal and written communication between researcher and funder - Automated notifications of report deadlines - The value of a peer support network - Support available to researchers from institutes, NHS trusts and other services to researchers for application and reporting - The importance of constructive feedback and rebuttal - Perceptions of how information is used by organisations and the need for clear communication on this - Clarification of the review and decision making process to allocate research funds and reporting requirements - Keeping the funder in the loop on research processes and progress via reporting | So you can basically do it formally or informally. So, when people within our group, for example, are thinking of writing a grant, we can share examples of previous grants, but the university also keeps some for the purposes of this mock exercises, but also to be able to share with people. P21  I think what would be really helpful but people are less willing to do it is to send you failed grants and to see where they’ve gone wrong as well as where things have gone well. P13  And again, they are responsive. I think funders are generally, there is somebody you can get in touch with, and they will answer your question. P8  Sometimes I think the automated reminders get a bit irritating. Having said that you need to plan and put time in, you don’t need, I don’t know, six months advance notice of when your final report’s due. P20  The biggest support is peer-to-peer support. P15  Yeah, so no, so you know, when you’ve got a great colleague, you receive a lot of wonderful support, you know, work collaboratively, and that’s really good, and the other tension is when somebody doesn’t, there’s a mismatch in expectations. P16  The RDS is incredibly helpful so they always provide fantastic feedback. P11  I think [institutional] support in terms of working out how to finance the grants that’s always been good. P3  Of course I always want more support and maybe more internal support to help me manage my projects. P9  There’s no consistency of people, and it just feels like you send emails and they go into a black hole sometimes. P4  If you want better applications you have to provide feedback, there is no way around it. P14  Feedback is really critical to working out what to do next time, whether that’s revamping the same grants, whether it’s ditching it and doing something completely different. P15  But I think the challenge is it's not always clear how this information is being used, whether it's being used, which goes back to that point about can we do this in a way that allows people to feel like the time that they’re spending on this stuff is actually well spent. P21  As I said, from the funder, I think clearer guidelines about expectations and how things are shortlisted. Because that’s where it's a bit of a black box at the moment, for us. Which is why we put in so much work to prepare for them. P5  But I think the funder should expect that any changes to the design, the protocol, anything else, should be reported, because I just think it increases sort of transparency, robustness and makes you think through the process. P5 |
