# Supplementary material for: An in-depth exploration of researcher experiences of time and effort involved in health and social care research funding in the UK: The need for changes
Source: PLoS One. 2023 Sep 21;18(9):e0291663. doi: 10.1371/journal.pone.0291663 (PMC10513312; doi:10.1371/journal.pone.0291663)
Supplement: S3 Table — Considerations are derived directly from interview data of researcher experiences. HEI = higher education institution; other refers to other research organisations, researchers or wider research community. (DOCX) [file pone.0291663.s003.docx]

**S3 Table. Summary table of considerations for reducing researcher burden and research waste from researchers experiences.**

| Number | Consideration | Relevant organisation |
| --- | --- | --- |
| 1 | Review the costs of running a research project to better understand what ‘value for money’ means in the current research landscape and to ensure these are not prohibitive to applying for funding | Funders, HEI |
| 2 | Be transparent about decision-making processes for funding allocation, including clear assessment criteria, strategic priorities, use of metrics, and chances of success and provide clear guidance to applicants on this | Funder |
| 3 | Provide reliable and fair peer review and decision-making through addressing potential biases and inconsistencies, selecting expert reviewers to match applications and considering alternative decision-making mechanisms such as blinding or randomisation | Funder |
| 4 | Explore how to reduce the time taken to make funding recommendations and communicate decisions in a timely manner | Funder |
| 5 | Provide constructive feedback on successful and unsuccessful applications and all submitted reports so that researchers can improve the quality of future documents | Funder |
| 6 | Consider the timelines of funding calls - including when the funding call opens in the calendar year and how long the call remains open | Funder |
| 7 | Align application and reporting practices and digital platforms across funders, institutions and other research organisations to share and re-use information and reduce duplication | Funder, HEI and Other |
| 8 | Review the usability and functionality of online systems and gather user feedback to continuously improve these systems. In particular, simplify registration for online application platforms, internal sign-off processes and processes for requesting extensions | Funder, HEI |
| 9 | Review and simplify application and reporting requirements to consider the amount of information requested, when it is requested, repetition and inconsistency and ensure processes are relevant and add value. | Funder |
| 10 | Consider how to better support preliminary application work for funding and completion of reporting requirements that falls outside of the funding period | Funder, HEI |
| 11 | Clearly state the purpose, expectations and benefits of reporting on research within the funding call and at the start of the research funding period | Funder |
| 12 | Be proportionate in the frequency and length of reports required throughout and beyond the funding period to increase the relevance and value of the information provided and avoid redundant/repetitive information | Funder |
| 13 | Provide institutional support for application and reporting requirements; including career and pastoral support for researchers | HEI |
| 14 | Provide explicit guidelines and expert support for developing research costings, including NHS costs, cost categories, indirect and direct costs, and acceptable changes in costs across stages of an application | Funder, HEI, Other |
| 15 | Set up good two-way channels of communication between researchers and funders, encourage verbal dialogue and respond to queries in a timely manner | Funder |
| 16 | Provide resources and training to researchers for all stages of the research funding and reporting processes, including access to previous applications, opportunities for networking and senior researchers mentoring those earlier in their careers | Funder, HEI, Other |
| 17 | Create a positive research culture by encouraging positive work-life balance and recognising the value of the efforts for each activity undertaken. This in turn will encourage more researchers to remain in research. | Funder, HEI, Other |

Note. Considerations are derived directly from interview data of researcher experiences. *HEI = higher education institution; **Other refers to other research-related organisations, researchers or wider research community
