# Supplementary material for: An in-depth exploration of researcher experiences of time and effort involved in health and social care research funding in the UK: The need for changes
Source: PLoS One. 2023 Sep 21;18(9):e0291663. doi: 10.1371/journal.pone.0291663 (PMC10513312; doi:10.1371/journal.pone.0291663)
Supplement: Attachment — Submitted filename: Response to reviewers.docx [file pone.0291663.s004.docx]

National Institute for Health and Care Research Coordinating Centre,

School of Healthcare Enterprise and Innovation,

University of Southampton,

Alpha House,

Enterprise Road,

Southampton,

SO16 7NS

30^th^ August 2023

Dear Dr Agwu

**Re: Revised Manuscript PONE-D-23-09154R1**

Thank you for your action letter on 30^th^ August 2023 regarding the manuscript PONE-D-23-09154R1 titled: “An in-depth exploration of researcher experiences of time and effort involved in health and social care research funding in the UK: The need for changes”. We have uploaded a marked up and clean copy of our revised manuscript. We have revised the formatting of the reference list. No additional references have been included and no references have been deleted. Please find below our responses to the comments itemised in your email. We hope that the manuscript now meets PLOS ONE’s publication criteria.

We look forward to hearing back from you.

Kind regards,

Katie Meadmore

Senior Reseach Fellow

NIHR, School of Healthcare Enterprise and Innovation, University of Southampton

**------------------------------------------------------------------------------------------------------------------------------------**

**Journal Requirements:**

Please review your reference list to ensure that it is complete and correct. If you have cited papers that have been retracted, please include the rationale for doing so in the manuscript text, or remove these references and replace them with relevant current references. Any changes to the reference list should be mentioned in the rebuttal letter that accompanies your revised manuscript. If you need to cite a retracted article, indicate the article’s retracted status in the References list and also include a citation and full reference for the retraction notice.

**Response:** Apologies for the formatting errors on the reference list. We have checked the reference list against the plos one guidelines and examples and believe that it is complete and correct. No cited papers have been retracted.

**Additional Editor Comments:**

The paper is greatly improved. However, some minor comments:

1. Signpost in the main paper where Figure 1 should appear, and refer to it in the narrative.

**Response**: Figure 1 is referred to in lines 179-181 and is signposted to appear around line 185.

2. Include supplementary files that you want added to the paper. For instance, you can take off the sociodemographic table, since you have provided detailed explanation of the features of participants. The COREQ checklist can be removed.

**Response**: Thank you for this advice. We have removed the COREQ checklist and demographic information from the supplemental material but kept the interview topic guide, summary table of themes and quotes and the table of recommendations.

3. Read through for grammatical errors and sentences that are not clear. Not so many of them, but please, read through.

**Response**: We have read through the document and amended the grammatical errors.
