# Supplementary material for: An in-depth exploration of researcher experiences of time and effort involved in health and social care research funding in the UK: The need for changes
Source: PLoS One. 2023 Sep 21;18(9):e0291663. doi: 10.1371/journal.pone.0291663 (PMC10513312; doi:10.1371/journal.pone.0291663)
Supplement: Peer review history

# Peer review: Decision Letter 0

**DOI:** 10.1371/journal.pone.0291663.r001

**Authors:** Prince Chiemeka Agwu

29 May 2023

PONE-D-23-09154An interview study exploring researcher experiences of time and effort involved in research funding in the UKPLOS ONE

Dear Dr. Meadmore,

Thank you for submitting your manuscript to PLOS ONE. After careful consideration, we feel that it has merit but does not fully meet PLOS ONE’s publication criteria as it currently stands. Therefore, we invite you to submit a revised version of the manuscript that addresses the points raised during the review process.

Please submit your revised manuscript by Jul 13 2023 11:59PM. If you will need more time than this to complete your revisions, please reply to this message or contact the journal office at plosone@plos.org. When you're ready to submit your revision, log on to https://www.editorialmanager.com/pone/ and select the 'Submissions Needing Revision' folder to locate your manuscript file.

Please include the following items when submitting your revised manuscript:

- A rebuttal letter that responds to each point raised by the academic editor and reviewer(s). You should upload this letter as a separate file labeled 'Response to Reviewers'.
- A marked-up copy of your manuscript that highlights changes made to the original version. You should upload this as a separate file labeled 'Revised Manuscript with Track Changes'.
- An unmarked version of your revised paper without tracked changes. You should upload this as a separate file labeled 'Manuscript'.

If you would like to make changes to your financial disclosure, please include your updated statement in your cover letter. Guidelines for resubmitting your figure files are available below the reviewer comments at the end of this letter.

If applicable, we recommend that you deposit your laboratory protocols in protocols.io to enhance the reproducibility of your results. Protocols.io assigns your protocol its own identifier (DOI) so that it can be cited independently in the future. For instructions see: https://journals.plos.org/plosone/s/submission-guidelines#loc-laboratory-protocols. Additionally, PLOS ONE offers an option for publishing peer-reviewed Lab Protocol articles, which describe protocols hosted on protocols.io. Read more information on sharing protocols at https://plos.org/protocols?utm_medium=editorial-email&utm_source=authorletters&utm_campaign=protocols.

We look forward to receiving your revised manuscript.

Kind regards,

Prince Chiemeka Agwu

Academic Editor

PLOS ONE

Journal Requirements:

When submitting your revision, we need you to address these additional requirements.

1. Please ensure that your manuscript meets PLOS ONE's style requirements, including those for file naming. The PLOS ONE style templates can be found at

https://journals.plos.org/plosone/s/file?id=wjVg/PLOSOne_formatting_sample_main_body.pdf and

https://journals.plos.org/plosone/s/file?id=ba62/PLOSOne_formatting_sample_title_authors_affiliations.pdf

2. We note that the grant information you provided in the ‘Funding Information’ and ‘Financial Disclosure’ sections do not match.

When you resubmit, please ensure that you provide the correct grant numbers for the awards you received for your study in the ‘Funding Information’ section.

3. Thank you for stating the following financial disclosure:

“This research was funded by the National Institute for Health and Care Research Coordinating Centre (NIHRCC), based at the University of Southampton, through its Research on Research Programme. The views and opinions expressed in the discussion are those of the authors and do not necessarily reflect those of the Department of Health and Social Care.”

Please state what role the funders took in the study.  If the funders had no role, please state: "The funders had no role in study design, data collection and analysis, decision to publish, or preparation of the manuscript."

If this statement is not correct you must amend it as needed.

Please include this amended Role of Funder statement in your cover letter; we will change the online submission form on your behalf.

4. Thank you for stating the following in the Competing Interests section:

“I have read the journal's policy and the authors of this manuscript have the following competing interests: All of the authors are employed by the School of Healthcare Enterprise and Innovation, University of Southampton and work within the National Institute for Health and Care Research (NIHR) Coordinating Centre. KF also holds a Post-Doctoral Fellowship funded by the NIHR.”

Please confirm that this does not alter your adherence to all PLOS ONE policies on sharing data and materials, by including the following statement: "This does not alter our adherence to  PLOS ONE policies on sharing data and materials.” (as detailed online in our guide for authors http://journals.plos.org/plosone/s/competing-interests).  If there are restrictions on sharing of data and/or materials, please state these. Please note that we cannot proceed with consideration of your article until this information has been declared.

Please include your updated Competing Interests statement in your cover letter; we will change the online submission form on your behalf.

5. In your Data Availability statement, you have not specified where the minimal data set underlying the results described in your manuscript can be found. PLOS defines a study's minimal data set as the underlying data used to reach the conclusions drawn in the manuscript and any additional data required to replicate the reported study findings in their entirety. All PLOS journals require that the minimal data set be made fully available. For more information about our data policy, please see http://journals.plos.org/plosone/s/data-availability.

Upon re-submitting your revised manuscript, please upload your study’s minimal underlying data set as either Supporting Information files or to a stable, public repository and include the relevant URLs, DOIs, or accession numbers within your revised cover letter. For a list of acceptable repositories, please see http://journals.plos.org/plosone/s/data-availability#loc-recommended-repositories. Any potentially identifying patient information must be fully anonymized.

Important: If there are ethical or legal restrictions to sharing your data publicly, please explain these restrictions in detail. Please see our guidelines for more information on what we consider unacceptable restrictions to publicly sharing data: http://journals.plos.org/plosone/s/data-availability#loc-unacceptable-data-access-restrictions. Note that it is not acceptable for the authors to be the sole named individuals responsible for ensuring data access.

We will update your Data Availability statement to reflect the information you provide in your cover letter.

6. Please include captions for your Supporting Information files at the end of your manuscript, and update any in-text citations to match accordingly. Please see our Supporting Information guidelines for more information: http://journals.plos.org/plosone/s/supporting-information.

7. Please review your reference list to ensure that it is complete and correct. If you have cited papers that have been retracted, please include the rationale for doing so in the manuscript text, or remove these references and replace them with relevant current references. Any changes to the reference list should be mentioned in the rebuttal letter that accompanies your revised manuscript. If you need to cite a retracted article, indicate the article’s retracted status in the References list and also include a citation and full reference for the retraction notice.

Additional Editor Comments:

In addition to comments from the reviewer, the authors should as well address the following queries:

“The aims of this study were to describe researchers’ experiences of the effort and burden involved in applying for research funding and fulfilling reporting requirements, and to provide recommendations on how these researcher experiences could be improved.” – here is the aim of the study but is not reflected in the title. I suggest rejigging the title a bit to capture the part where you want the experiences of the researchers to improve. As the topic is presented, it appears you just want to describe the experiences and leave it at that.

The first sentence in the introduction does not connect well with the substance of the research.

As part of the researchers’ burden you captured in the introduction, navigating implementation of findings was missing. I would not like to see people read this paper and think researchers are only interested in the outcomes that benefit their academic records (e.g., publications, technical reports, op-eds, etc.) and the traditional academic deliverables for funders.

It will be great to describe the area and context where the research was conducted. It should be part of the results.

It is important to go back to COREQ framework and align your research reporting pattern to fit with most of its expectations.

Under data collection, kindly explain “… and helped reduce potential interviewer bias”.

Was there a pretest of the study tool?

The results section is too lengthy. We want people to read our papers. My advice is to do some merging of the themes. I would not want to point to themes that could be merged because I would not want to interrupt what the authors have in mind. So, try to merge similar ideas and bring the themes to four. You can have very few subthemes under the main themes. There is a lot of information that still needs to be organised.

I would caution against having the recommendations as part of the results because they are not fully evidence driven. How about taking the recommendations as a part of the discussion and add the table somewhere at the base of the discussion. Also, the recommendations can be merged to avoid repetitions. I will want to advice again that the authors can produce a table that shows the issues that these recommendations are addressing, after they have been streamlined, purging off repetitions.

Also, since you have a lot of findings, I will recommend a good figure ties your findings together, making the evidence punchy, and providing a model that can easily be reflected on once this issue comes to mind.

Try to indent quotes with explainers in-between. So many words can be saved from the results. As the manuscript is currently, it is too long and will be a turn off for people to read. You can as well cut the paragraphs that are too long so that this paper can be reader-friendly.

I want to see the connections of these findings to the implementation of research findings in policies, programmes, and improving communities. Even if the data do not have such information, I am encouraging the authors to think through establishing such connections by making inferences using available evidence and reflecting such in the discussion section.

When you say research environment, are you referring to the European research environment alone?

Your usage of “research culture” as a concept is unclear. It needs an explainer.

The research questions guiding the study should be clearly stated in the introduction.

[Note: HTML markup is below. Please do not edit.]

Reviewers' comments:

Reviewer's Responses to Questions

**Comments to the Author**

1. Is the manuscript technically sound, and do the data support the conclusions?

The manuscript must describe a technically sound piece of scientific research with data that supports the conclusions. Experiments must have been conducted rigorously, with appropriate controls, replication, and sample sizes. The conclusions must be drawn appropriately based on the data presented. 

Reviewer #1: Yes

**********

2. Has the statistical analysis been performed appropriately and rigorously? 

Reviewer #1: Yes

**********

3. Have the authors made all data underlying the findings in their manuscript fully available?

The PLOS Data policy requires authors to make all data underlying the findings described in their manuscript fully available without restriction, with rare exception (please refer to the Data Availability Statement in the manuscript PDF file). The data should be provided as part of the manuscript or its supporting information, or deposited to a public repository. For example, in addition to summary statistics, the data points behind means, medians and variance measures should be available. If there are restrictions on publicly sharing data—e.g. participant privacy or use of data from a third party—those must be specified.

Reviewer #1: Yes

**********

4. Is the manuscript presented in an intelligible fashion and written in standard English?

PLOS ONE does not copyedit accepted manuscripts, so the language in submitted articles must be clear, correct, and unambiguous. Any typographical or grammatical errors should be corrected at revision, so please note any specific errors here.

Reviewer #1: Yes

**********

5. Review Comments to the Author

Please use the space provided to explain your answers to the questions above. You may also include additional comments for the author, including concerns about dual publication, research ethics, or publication ethics. (Please upload your review as an attachment if it exceeds 20,000 characters)

Reviewer #1: Thanks for taking up the challenge to conduct this study. This is such an important study.

Please see my review:

SAMPLING TECHNIQUE

The sampling technique was explained, however, it was not named. Could this purposive or convenient? Please name it properly.

RESULT SECTION

173 and most of the result section. The authors should consider the use of "participants" instead of interviewees.

288: The authors commented on participants’ responses to a deadline. I was wondering if this meaningful information belongs to the Theme of “Implications for Research Culture” or “Timeframes for Research”?

For instance, in 333 and 290, funding calls very close to holidays like Christmas were described.

Is this a meaningful convergence?

379: The authors may consider merging this theme “developing the application” to the previous theme on “Timeline”. The meaning that I am exploring is essentially that of “time and effort”. Many participants are describing that it takes more time to develop an application. Developing applications does not seem to be the meaningful information here, what is meaningful is the "time" they invest.

664. The authors should consider numbering the recommendations in the table.

664. The authors should also organise the recommendations based on their three categories in a table. For instance, Recommendations for Funders, HEIs and Other Research-Related Organizations.

Trustworthiness:

The authors should tell the scientific community how they ensured Trustworthiness. Although, they provided "Reflexivity Statement" which is great. However, considering that this study is about "researchers experiences with research funding" is being conducted by researchers who also apply for funding and the use of inductive thematic analysis; I would recommend a stronger statement or process of trustworthiness to enable the scientific community to see the paper as credible and dependable and void of any bias.

Limitations of the Study

The authors were economical with their limitations. They did not explain much about the limitation of this study. For instance, the participants of this study were not racially diverse. 85% White/British (4 participants did not disclose their race). Essentially, the study had only participants who identified as White from England, Scotland and Wales. The UK has many other researchers who are British but are Black and Ethnic Minorities (BAME). Non-inclusion of others like Black may not be the fault of the authors if they use purposive sampling, however, it should be acknowledged transparently.

Is there any reason for exclusion? State.

Do they think that the researchers from BAME would have different experiences or opinions? Why?

It is important to acknowledge the diversity issue in the study and encourage other studies to consider this.

**********

6. PLOS authors have the option to publish the peer review history of their article (what does this mean?). If published, this will include your full peer review and any attached files.

If you choose “no”, your identity will remain anonymous but your review may still be made public.

**Do you want your identity to be public for this peer review?** For information about this choice, including consent withdrawal, please see our Privacy Policy.

Reviewer #1: No

**********

[NOTE: If reviewer comments were submitted as an attachment file, they will be attached to this email and accessible via the submission site. Please log into your account, locate the manuscript record, and check for the action link "View Attachments". If this link does not appear, there are no attachment files.]

While revising your submission, please upload your figure files to the Preflight Analysis and Conversion Engine (PACE) digital diagnostic tool, https://pacev2.apexcovantage.com/. PACE helps ensure that figures meet PLOS requirements. To use PACE, you must first register as a user. Registration is free. Then, login and navigate to the UPLOAD tab, where you will find detailed instructions on how to use the tool. If you encounter any issues or have any questions when using PACE, please email PLOS at figures@plos.org. Please note that Supporting Information files do not need this step.

# Author comment: Author response to Decision Letter 0

**DOI:** 10.1371/journal.pone.0291663.r002

24 Aug 2023

Additional Editor Comments:

1.1: “The aims of this study were to describe researchers’ experiences of the effort and burden involved in applying for research funding and fulfilling reporting requirements, and to provide recommendations on how these researcher experiences could be improved.” – here is the aim of the study but is not reflected in the title. I suggest rejigging the title a bit to capture the part where you want the experiences of the researchers to improve. As the topic is presented, it appears you just want to describe the experiences and leave it at that.

Response: As suggested in 1.9, we have removed the table of recommendations from the results section and added it as a supporting information file. The primary aim of this study was to describe researchers’ experiences and in doing so we have then gained better understanding or inferred how these experiences could be improved; as such, we have removed the recommendations section from the main results section and have emphasized this in the title and aims.

1.2: The first sentence in the introduction does not connect well with the substance of the research.

Response: This has now been changed. 

1.3: As part of the researchers’ burden you captured in the introduction, navigating implementation of findings was missing. I would not like to see people read this paper and think researchers are only interested in the outcomes that benefit their academic records (e.g., publications, technical reports, op-eds, etc.) and the traditional academic deliverables for funders.

Response: Thank you for pointing this out, it certainly was not our intention to suggest that researchers are only interested in outcomes that benefit their academic record. We have made this more explicit in the introduction.

1.4: It will be great to describe the area and context where the research was conducted. It should be part of the results.

Response: This has been added to the methods under the Qualitative approach and Reflexivity statement sections.

1.5: It is important to go back to COREQ framework and align your research reporting pattern to fit with most of its expectations.

Response: We did try to ensure that all the domains on the COREQ checklist were reported in the paper. We have now included the checklist (and where the information can be found for each item) as a supporting information file (S1 Table).

1.6: Under data collection, kindly explain “… and helped reduce potential interviewer bias”.

Response: A qualifier has been added: “the topic guide was used to ensure all interviews followed a structure which helped reduce potential interviewer bias by ensuring that the same questions were asked and addressed”.

1.7: Was there a pretest of the study tool?

Response: The interview schedule was reviewed by the members of NIHR and the team working on the Independent Review of Research Bureaucracy to ensure that the questions were relevant to funders and the wider research sector and remained objective and generalisable. Furthermore, following the first 7 interviews, the topic guide was reviewed and refined. This has been made explicit in the text. This has been amended in the Interview guide section.

1.8: The results section is too lengthy. We want people to read our papers. My advice is to do some merging of the themes. I would not want to point to themes that could be merged because I would not want to interrupt what the authors have in mind. So, try to merge similar ideas and bring the themes to four. You can have very few subthemes under the main themes. There is a lot of information that still needs to be organised.

Response: Thank you for your advice. As noted by you and reviewer 1 the themes do interlink and there was convergence across themes. We have reviewed and revised the themes, resulting in a final five themes. It would not have made thematical sense for the data to be combined further and would not be a true reflection of participants varied views and experiences. We have revised and organised all the information and have reduced this section by ~2500 words. Whilst we appreciate that the results section is still relatively long, we feel confident that this is reasonable for a qualitative study of this size. 

1.9: I would caution against having the recommendations as part of the results because they are not fully evidence driven. How about taking the recommendations as a part of the discussion and add the table somewhere at the base of the discussion. Also, the recommendations can be merged to avoid repetitions. I will want to advice again that the authors can produce a table that shows the issues that these recommendations are addressing, after they have been streamlined, purging off repetitions.

Response: Thank you for your suggestion about the recommendations. We were unsure as to whether to include them in the first submission and your comment has confirmed that these would be better as supporting information files (see S5 Table). The table has been amended in line with the reviewer’s comment (2.5). Recommendation points are woven throughout the discussion and the S5 Table is referred to in this section.

1.10: Also, since you have a lot of findings, I will recommend a good figure ties your findings together, making the evidence punchy, and providing a model that can easily be reflected on once this issue comes to mind.

Response: We have now included a figure that illustrates the themes from this study and shows the links between the themes.

1.11: Try to indent quotes with explainers in-between. So many words can be saved from the results. As the manuscript is currently, it is too long and will be a turn off for people to read. You can as well cut the paragraphs that are too long so that this paper can be reader-friendly.

Response: The results section has been reduced by 7 pages/~2500 words. In addition to reducing the themes from 7 to 5, we have reduced the length or removed some quotes and reduced text from each theme. The longer quotes have been further indented but we have kept some shorter quotes within the text as we feel that using the participants words helps to illustrate contexts and interpretation. We hope that you feel that the revised version of the results is more reader-friendly. 

1.12: I want to see the connections of these findings to the implementation of research findings in policies, programmes, and improving communities. Even if the data do not have such information, I am encouraging the authors to think through establishing such connections by making inferences using available evidence and reflecting such in the discussion section.

Response: Thank you for this comment. We did try to link our interpretations to previous work; however, we have now made additional explicit links to UK strategies and policies as well. 

1.13: When you say research environment, are you referring to the European research environment alone?

Response: The participants were all UK researchers who received UK research funding and so research environment refers to the UK research environment. This has been made more explicit in the implications for work-life balance theme.

1.14: Your usage of “research culture” as a concept is unclear. It needs an explainer.

Response: Thank you for this comment. We refer to research culture as the expected behaviours, values, and attitudes of research communities and the effects that this has on researchers. Based on this comment and to better capture the issues described in the previously named ‘implications to research culture’ theme, we have changed the theme name to ‘implications to work-life balance’. We have also described what we mean when we refer to research culture in the discussion and in the newly named ‘implications to work-life balance’ theme. 

1.15: The research questions guiding the study should be clearly stated in the introduction.

 Response: This has been added in the introduction. 

Reviewers' comments:

Reviewer #1: Thanks for taking up the challenge to conduct this study. This is such an important study.

Response: Thank you for your comment. We agree and feel that this is an important and timely topic.

2.1: SAMPLING TECHNIQUE

The sampling technique was explained, however, it was not named. Could this purposive or convenient? Please name it properly.

Response: Apologies for this oversight, this has now been added in the Methods: Recruitment section.

RESULT SECTION

2.2: 173 and most of the result section. The authors should consider the use of "participants" instead of interviewees.

Response: This has been changed throughout the paper.

2.3.: 288: The authors commented on participants’ responses to a deadline. I was wondering if this meaningful information belongs to the Theme of “Implications for Research Culture” or “Timeframes for Research”? For instance, in 333 and 290, funding calls very close to holidays like Christmas were described. Is this a meaningful convergence?

Response: Thank you for this insightful comment. We agree that there are links and convergences across the themes, and in this case, it was confusing that the different concepts were described in two themes. In line with yours and the editor’s comments we have now revised our themes and have also included a figure (Fig 1) to help illustrate the connections between the themes. 

2.4: 379: The authors may consider merging this theme “developing the application” to the previous theme on “Timeline”. The meaning that I am exploring is essentially that of “time and effort”. Many participants are describing that it takes more time to develop an application. Developing applications does not seem to be the meaningful information here, what is meaningful is the "time" they invest.

Response: Thank you for this suggestion. We had originally grouped these themes together in a bigger theme on time and effort involved in research. However, we were worried that this theme was too large and so separated these two concepts. We agree that in doing this, we lost the main concept of these themes. In line with this and the editor comments, these themes have been merged.

2.5: 664. The authors should consider numbering the recommendations in the table.

Response: We have added numbers to the recommendation table (see S5 Table)

2.6: 664. The authors should also organise the recommendations based on their three categories in a table. For instance, Recommendations for Funders, HEIs and Other Research-Related Organizations.

Response: We have now added the funder, HEI and other categories to the recommendation table (see S5 Table)

2.7: Trustworthiness: The authors should tell the scientific community how they ensured Trustworthiness. Although, they provided "Reflexivity Statement" which is great. However, considering that this study is about "researchers experiences with research funding" is being conducted by researchers who also apply for funding and the use of inductive thematic analysis; I would recommend a stronger statement or process of trustworthiness to enable the scientific community to see the paper as credible and dependable and void of any bias.

Response: Thank you for this comment. We have added information about the context in which this work took place, who we are and the experience of the team, all of which we feel adds to the credibility of the team and the findings. We have also emphasised that the interview questions and themes were discussed with the wider team and senior colleagues to ensure that the data were data driven and not biased. This has been added to the methods under the Qualitative approach, Interview guide and Reflexivity statement sections.

2.8: Limitations of the Study: The authors were economical with their limitations. They did not explain much about the limitation of this study. For instance, the participants of this study were not racially diverse. 85% White/British (4 participants did not disclose their race). Essentially, the study had only participants who identified as White from England, Scotland and Wales. The UK has many other researchers who are British but are Black and Ethnic Minorities (BAME). Non-inclusion of others like Black may not be the fault of the authors if they use purposive sampling, however, it should be acknowledged transparently. Is there any reason for exclusion? State. Do they think that the researchers from BAME would have different experiences or opinions? Why? It is important to acknowledge the diversity issue in the study and encourage other studies to consider this.

Response: Thank you for this comment. We certainly hadn’t intended on being economical with our limitations and agree that further discussion on this limitation is an omission from this section. As such, we have included additional text on the diversity in health and social care research. In addition, on reflection we feel that in wanting to retain confidentiality of participants we did not clearly report participants’ demographics and so we have also amended this section to make the demographics of our participants clearer.

# Editor report: Decision Letter 1

**DOI:** 10.1371/journal.pone.0291663.r003

**Authors:** Prince Chiemeka Agwu

30 Aug 2023

PONE-D-23-09154R1An in-depth exploration  of researcher experiences of time and effort involved in health and social care research funding in the UK: The need for changesPLOS ONE

Dear Dr. Meadmore,

Thank you for submitting your manuscript to PLOS ONE. After careful consideration, we feel that it has merit but does not fully meet PLOS ONE’s publication criteria as it currently stands. Therefore, we invite you to submit a revised version of the manuscript that addresses the points raised during the review process.

Please submit your revised manuscript by Oct 14 2023 11:59PM. If you will need more time than this to complete your revisions, please reply to this message or contact the journal office at plosone@plos.org. When you're ready to submit your revision, log on to https://www.editorialmanager.com/pone/ and select the 'Submissions Needing Revision' folder to locate your manuscript file.

Please include the following items when submitting your revised manuscript:

- A rebuttal letter that responds to each point raised by the academic editor and reviewer(s). You should upload this letter as a separate file labeled 'Response to Reviewers'.
- A marked-up copy of your manuscript that highlights changes made to the original version. You should upload this as a separate file labeled 'Revised Manuscript with Track Changes'.
- An unmarked version of your revised paper without tracked changes. You should upload this as a separate file labeled 'Manuscript'.

If you would like to make changes to your financial disclosure, please include your updated statement in your cover letter. Guidelines for resubmitting your figure files are available below the reviewer comments at the end of this letter.

If applicable, we recommend that you deposit your laboratory protocols in protocols.io to enhance the reproducibility of your results. Protocols.io assigns your protocol its own identifier (DOI) so that it can be cited independently in the future. For instructions see: https://journals.plos.org/plosone/s/submission-guidelines#loc-laboratory-protocols. Additionally, PLOS ONE offers an option for publishing peer-reviewed Lab Protocol articles, which describe protocols hosted on protocols.io. Read more information on sharing protocols at https://plos.org/protocols?utm_medium=editorial-email&utm_source=authorletters&utm_campaign=protocols.

We look forward to receiving your revised manuscript.

Kind regards,

Prince Chiemeka Agwu

Academic Editor

PLOS ONE

Journal Requirements:

Please review your reference list to ensure that it is complete and correct. If you have cited papers that have been retracted, please include the rationale for doing so in the manuscript text, or remove these references and replace them with relevant current references. Any changes to the reference list should be mentioned in the rebuttal letter that accompanies your revised manuscript. If you need to cite a retracted article, indicate the article’s retracted status in the References list and also include a citation and full reference for the retraction notice.

Additional Editor Comments:

The paper is greatly improved. However, some minor comments:

1. Signpost in the main paper where Figure 1 should appear, and refer to it in the narrative.

2. Include supplementary files that you want added to the paper. For instance, you can take off the sociodemographic table, since you have provided detailed explanation of the features of participants. The COREQ checklist can be removed.

3. Read through for grammatical errors and sentences that are not clear. Not so many of them, but please, read through.

[Note: HTML markup is below. Please do not edit.]

[NOTE: If reviewer comments were submitted as an attachment file, they will be attached to this email and accessible via the submission site. Please log into your account, locate the manuscript record, and check for the action link "View Attachments". If this link does not appear, there are no attachment files.]

While revising your submission, please upload your figure files to the Preflight Analysis and Conversion Engine (PACE) digital diagnostic tool, https://pacev2.apexcovantage.com/. PACE helps ensure that figures meet PLOS requirements. To use PACE, you must first register as a user. Registration is free. Then, login and navigate to the UPLOAD tab, where you will find detailed instructions on how to use the tool. If you encounter any issues or have any questions when using PACE, please email PLOS at figures@plos.org. Please note that Supporting Information files do not need this step.

# Author comment: Author response to Decision Letter 1

**DOI:** 10.1371/journal.pone.0291663.r004

30 Aug 2023

Additional Editor Comments:

The paper is greatly improved. However, some minor comments:

1. Signpost in the main paper where Figure 1 should appear, and refer to it in the narrative.

Response: Figure 1 is referred to in lines 179-181 and is signposted to appear around line 185.

2. Include supplementary files that you want added to the paper. For instance, you can take off the sociodemographic table, since you have provided detailed explanation of the features of participants. The COREQ checklist can be removed.

Response: Thank you for this advice. We have removed the COREQ checklist and demographic information from the supplemental material but kept the interview topic guide, summary table of themes and quotes and the table of recommendations.

3. Read through for grammatical errors and sentences that are not clear. Not so many of them, but please, read through.

Response: We have read through the document and amended the grammatical errors.

Attachment Submitted filename: Response to reviewers.docx Click here for additional data file.

# Editor report: Decision Letter 2

**DOI:** 10.1371/journal.pone.0291663.r005

**Authors:** Prince Chiemeka Agwu

4 Sep 2023

An in-depth exploration  of researcher experiences of time and effort involved in health and social care research funding in the UK: The need for changes

PONE-D-23-09154R2

Dear Dr. Meadmore,

We’re pleased to inform you that your manuscript has been judged scientifically suitable for publication and will be formally accepted for publication once it meets all outstanding technical requirements.

Within one week, you’ll receive an e-mail detailing the required amendments. When these have been addressed, you’ll receive a formal acceptance letter and your manuscript will be scheduled for publication.

An invoice for payment will follow shortly after the formal acceptance. To ensure an efficient process, please log into Editorial Manager at http://www.editorialmanager.com/pone/, click the 'Update My Information' link at the top of the page, and double check that your user information is up-to-date. If you have any billing related questions, please contact our Author Billing department directly at authorbilling@plos.org.

If your institution or institutions have a press office, please notify them about your upcoming paper to help maximize its impact. If they’ll be preparing press materials, please inform our press team as soon as possible -- no later than 48 hours after receiving the formal acceptance. Your manuscript will remain under strict press embargo until 2 pm Eastern Time on the date of publication. For more information, please contact onepress@plos.org.

Kind regards,

Prince Chiemeka Agwu

Academic Editor

PLOS ONE

Additional Editor Comments (optional):

Reviewers' comments:

# Editor report: Acceptance letter

**DOI:** 10.1371/journal.pone.0291663.r006

**Authors:** Prince Chiemeka Agwu

13 Sep 2023

PONE-D-23-09154R2 

An in-depth exploration of researcher experiences of time and effort involved in health and social care research funding in the UK: The need for changes 

Dear Dr. Meadmore:

I'm pleased to inform you that your manuscript has been deemed suitable for publication in PLOS ONE. Congratulations! Your manuscript is now with our production department. 

If your institution or institutions have a press office, please let them know about your upcoming paper now to help maximize its impact. If they'll be preparing press materials, please inform our press team within the next 48 hours. Your manuscript will remain under strict press embargo until 2 pm Eastern Time on the date of publication. For more information please contact onepress@plos.org.

If we can help with anything else, please email us at plosone@plos.org. 

Thank you for submitting your work to PLOS ONE and supporting open access. 

Kind regards, 

PLOS ONE Editorial Office Staff

on behalf of

Dr. Prince Chiemeka Agwu 

Academic Editor

PLOS ONE
